# Supplementary material for: N-acetylchitohexaose confers resistance to Pseudomonas aeruginosa infection in the silkworm, Bombyx mori
Source: Infect Immun. 2025 Nov 18;93(12):e00385-25. doi: 10.1128/iai.00385-25 (PMC12707137; doi:10.1128/iai.00385-25)
Supplement: Supplemental Material — Fig. S1 to S5; Table S1 caption. [file iai.00385-25-s0001.pdf]

Supplemental Table & Figure

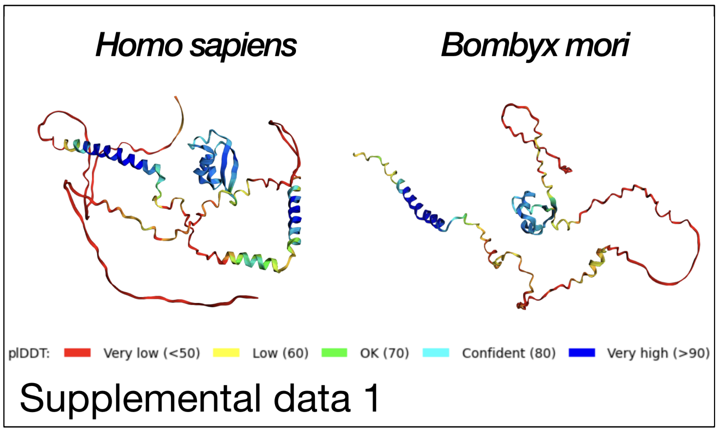

Supplementary Figure S1. AlphaFold-predicted structures and confidence scores for LysM-containing proteins from *Homo sapiens* and *Bombyx mori*.

Predicted structures of human LYSMD3 and its putative *B. mori* homolog (XP_004933441.1) were generated using AlphaFold and visualized with per-residue confidence scores (pLDDT). Regions with high prediction confidence (pLDDT > 90, shown in dark blue) were concentrated within the LysM domain in both species, supporting the structural comparison presented in Figure 1B. Regions outside the LysM domain showed lower confidence scores (light blue to red) and were excluded from RMSD-based comparative analysis.

Supplemental Table 1

The annotated list of differentially expressed genes (DEGs) is provided in Supplementary Table 1 (attached as a separate file due to file size limitations).

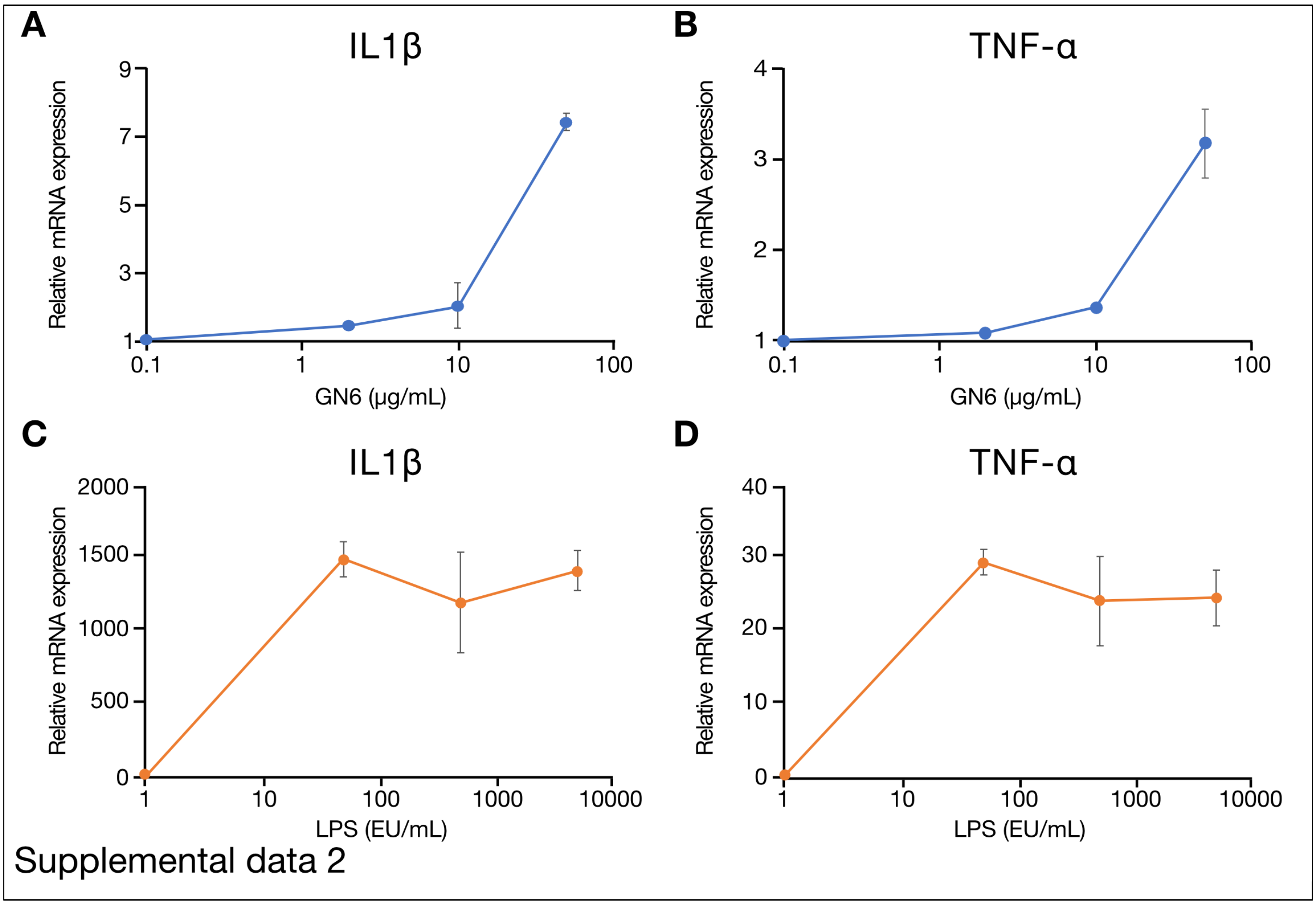

Supplementary Figure S**2. GN6 induces the expression of proinflammatory cytokine genes in RAW264.7 cells.**

Mouse macrophage RAW264.7 cells were stimulated with GN6 (A, B) or lipopolysaccharide (LPS, C, D) for 2 h. Gene expression levels of the proinflammatory cytokines *Il1b* and *Tnf* were analyzed by qRT-PCR and normalized to the internal control gene *Rps18*. Results are presented as fold changes relative to unstimulated controls. GN6 induced dose-dependent upregulation of both cytokine genes, whereas LPS induced a strong response in both *Il1b* and *Tnf*. Data represent mean ± SD (n = 3 each).

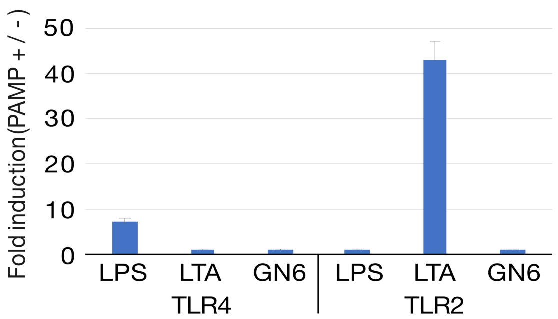

Supplemental data 3

Supplementary Figure S3. Evaluation of GN6-induced activation of human TLR2 and TLR4 pathways using HEK293 reporter cells.

HEK293 reporter cells expressing either human TLR4/CD14/MD2 or TLR2, along with a firefly luciferase gene under the control of an NF-κB response element, were used to assess GN6-induced pathway activation. Cells were stimulated for 6 h with GN6 (5 µg/mL), lipopolysaccharide (LPS; 1 µg/mL) as a positive control for TLR4, or lipoteichoic acid (LTA; 1 µg/mL) as a positive control for TLR2. Luciferase activity was quantified using the Dual-Luciferase Reporter Assay System, and firefly luciferase signals were normalized to Renilla luciferase. Results are expressed as fold induction relative to unstimulated controls. Data represent the mean ± SD of three replicates.

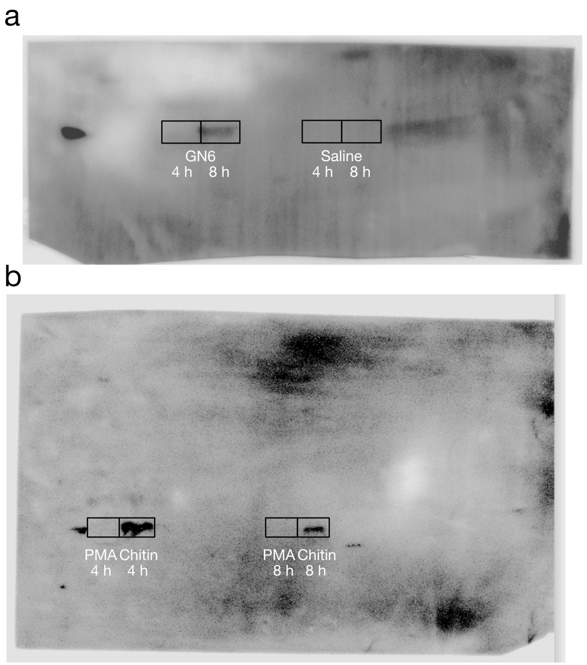

Supplemental data 4

Supplementary Figure S4. Full Western blot images for detection of Cecropin B in silkworm hemolymph following treatment with GN6, chitin, or PMA.

(a, b) Fifth-instar day-2 *Bombyx mori* larvae were injected with GN6 (2.5 µg/larva), chitin (2.5 µg/larva), PMA (0.5 ng/larva), or saline, and hemolymph samples were collected at 4 h and 8 h post-injection at 27°C. Cecropin B protein levels in the hemolymph were analyzed by western blotting.

(a) Blot showing samples from larvae treated with GN6 and saline.

(b) Blot showing samples from larvae treated with PMA and chitin.

The boxed areas indicate regions cropped and presented in the main text (Figure 3C).

Supplemental data 5

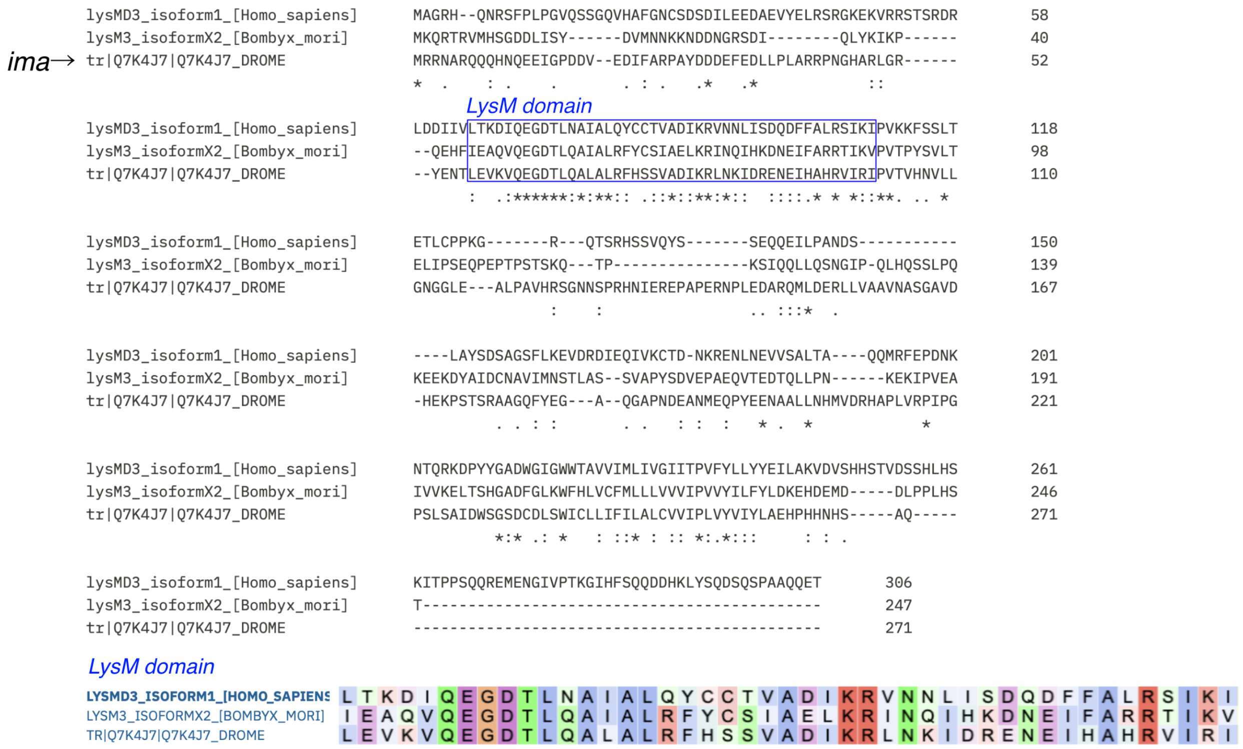

Supplemental Figure S5. Cross-species sequence alignment of LYSMD3 homologs in *Homo sapiens*, *Bombyx mori*, and *Drosophila melanogaster*.

Amino acid sequences of LYSMD3 homologs from humans (*LYSMD3_isoform1*), silkworms (*lysM3_isoformX2*), and fruit flies (*Q7K4J7_DROME*, corresponding to *ima*) were aligned using Clustal Omega (EMBL-EBI). The alignment shows that the LysM domain region is highly conserved across the three species, with no major divergence observed in the core motif.
